# Role of CAPE in reducing oxidative stress in animal models with traumatic brain injury

**DOI:** 10.1016/j.amsu.2020.07.036

**Published:** 2020-07-22

**Authors:** Rizha Anshori Nasution, Andi Asadul Islam, Mochammad Hatta, Prihantono, Agus Turchan, Nasrullah, Muhammad Faruk

**Affiliations:** a Department of Neurosurgery, Pelamonia Hospital, Makassar, Indonesia; b Doctoral Program of Biomedical Sciences, Faculty of Medicine, Hasanuddin University, Makassar, Indonesia; c Department of Neurosurgery, Faculty of Medicine, Hasanuddin University, Makassar, Indonesia; d Clinical Microbiologist Program, Faculty of Medicine, Hasanuddin University, Makassar, Indonesia; e Department of Surgery Faculty of Medicine, Hasanuddin University, Makassar, Indonesia; f Departement of Neurosurgery, Faculty of Medicine, Airlangga University, Surabaya, Indonesia

**Keywords:** Traumatic brain injury, F2-isoprostane, Oxidative stress

## Abstract

**Introduction:**

The central nervous system (CNS) is the most metabolically active organ characterized by high oxygen demand and relatively low anti-oxidative activity, which makes neurons and glia highly susceptible to damage by reactive oxygen and nitrogen byproducts as well as neurodegeneration. Free radicals are associated with secondary injuries that occur after a primary brain injury. Some of these free radical products include F2-Isoprostane (F2-IsoPs), malondialdehyde (MDA), 4-hydroxy-2-nonenal (4-HNE) and acrolein.

**Methods:**

In this study we measured serum F2-IsoPs levels as markers of free radical activity in 10–12 week-old male Sprague-Dawley rats weighing 200–300 g, all rats (n = 10) subjected with a head injury according to the modified marmourou model, then divided into 2 groups, one group treated with CAPE (Caffeic Acid Phenethyl Ester) (n = 5) and the other not treated with CAPE (n = 5), serum levels in the two groups were compared starting from day-0 (before brain injury), day-4 and day-7.

**Results:**

We found lower F2-IsoPs levels in the group that received the CAPE treatment compared to the group that did not receive the CAPE treatment.

**Conclusion:**

CAPE is capable of significantly reducing oxidative stress in brain injury.

## Highlights

• The central nervous system (CNS) is the most metabolically active organ characterized by high oxygen demand and relatively low anti-oxidative activity.
• Free radicals are associated with secondary injuries that occur after a primary brain injury.
• Caffeic Acid Phenethyl Ester (CAPE) administration in a rat model with brain injury can reduce the formation of F2-Isoprostane (F2-IsoPs) as an indicator of oxidative stress in blood serum post-trauma.

## 1 Introduction

Oxidative stress is a condition caused by a shift between pro-oxidants to antioxidants that can create organic damage [1]. Pro-oxidant by definition is a free radical, single or grouped atoms can be either paired with electrons or unpaired, while antioxidants are chemical compounds that can bind to free radicals so as not to cause cell damage [1,2]. Oxidative stress can be caused by several conditions such as inflammation, carcinogenesis, neurodegeneration, growth process and trauma to the CNS [[1], [2], [3]].

The brain is a part of the CNS. It is very susceptible to oxidative stress due its high oxygen demand. About 20–30% of the oxygen of the inspired oxygen, is consumed by brain, its high levels of polyunsaturated fatty acids (PUFA) [4], as well as its weak antioxidant defense system [5]. This susceptibility is increased in several conditions especially in brain injury due to damage of the blood-brain barrier (BBB), release of neurotransmitters such as glutamate, mitochondrial dysfunction and increased production of reactive oxygen species (ROS) [6]. Over production of ROS is what will cause oxidative stress in cases like brain injury [[5], [6], [7]].

**Fig. 1:**
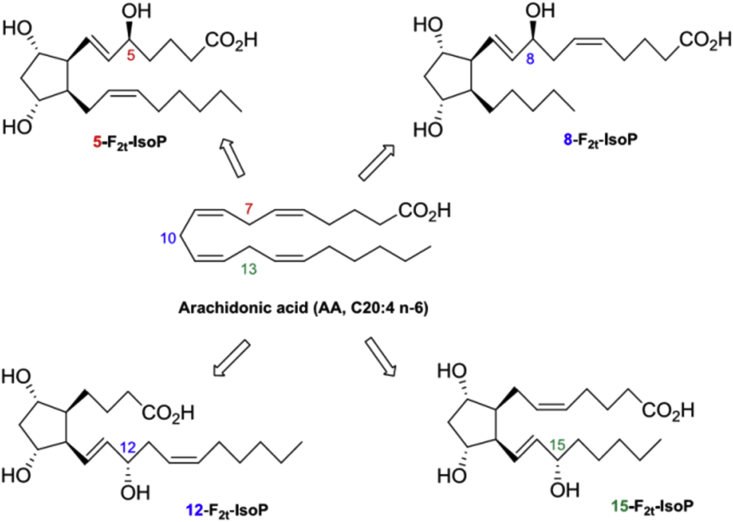
F2-isoprostane formation from arachidonic acid, which Leads to four F2-Isoprostane isomers [10].

F2-Isoprostane (F2-IsoPs) is a unique series of prostaglandin-like compounds that are formed in vivo and in vitro through a non-enzymatic peroxidation mechanism mediated by free radicals of arachidonic acid (Fig. 1) [3,8]. This compound was first discovered by Morrow and colleagues who explained the formation of F2-IsoPs from arachidonic acid [3,[8], [9], [10]].

Roberts and Marrow explained that there are six main advantages to using F2-IsoPs as a marker for oxidative damage in vivo [3,8,9,11,12].

## 2 F2 IsoPs are extremely stable compounds

2. F2-IsoPs are the most specific markers of lipid peroxidation in vivo compared to other lipid peroxidation markers, such as malondialdehyde (MDA) or lipid hydroperoxide.
3. F2-IsoPs are ready to be detected in all types of biological tissue and body fluids by the GC/NICI-MS method, which allows researchers to be able to determine a normal range or reference which is valuable for clinical studies in various conditions.
4. It has been well proven that F2-IsoPs levels are elevated in various animal models of oxidative stress.
5. F2-IsoPs levels are modulated by antioxidant system status.
6. F2-IsoPs levels are not influenced by diet.

Since then, the quantification of F2-IsoPs has been considered as one of the most accurate approaches for assessing oxidative damage in vitro [3,4,13]. F2-IsoPs are more than just a biomarker or a monitor for physiological processes, but can also act as a lipid mediator in vasoconstriction and platelet aggregation and can play a role in intracellular signaling through activation of prostanoid receptors [4,14,15].

CAPE is obtained from propolis honey bee through extraction [16], the structure of CAPE contains catechol which is a powerful antioxidant [17]. Propolis has long been used for many years as traditional medicine [18]. The antioxidant effect of propolis which contain CAPE is stronger if compared to propolis without CAPE [17,18]. CAPE has also been reported to inhibit prostaglandin (PG) and leukotriene synthesis. Prostaglandins (PG) plays an important role in the progression of inflammation, CAPE's antioxidant activity is shown in its role in inhibiting the release of arachidonic acid and COX-1 and COX-2 activities [[19], [20], [21]].

## 3 Materials and Methods

This study examines the oxidative stress caused by brain trauma by evaluating the time course of the accumulation of F2-IsoPs in the serum, and comparing the responses between both groups; the group treated with CAPE and the group not treated with CAPE.

### 3.1 Surgical procedure

This study was approved by the ethics commission of Faculty of Medicine, Hasanuddin University, license number: 771/UN4.6.4.5.31/PP36/2019. Surgical procedures were performed aseptically. Ten male Sprague-Dawley rats free of viruses and other pathogens, (more than 2 months-old, weighing 280–300 g) had adequate access to standard food (Comfeed AD-2) and water until the time of the study. Rats were placed in two groups: (1) head injury with the CAPE treatment, (2) head injury without the CAPE treatment.

Rats were induced with ketamine which had been diluted at a dose of 3-10 mg/kg, a head injury in accordance with the modified Marmarou model was conducted [21,22]. The surgical procedure began with the aseptic procedure using povidone iodine, then a coronal incision was made through the center of the skull and then a burr holes was performed using a high-speed drill until the duramater was exposed; a 1.5 cm long craniectomy was done, then a mass of 20 g was dropped from a height of 20 cm by passing through a tube posing as a transport medium, the area with the exposed duramater was placed just below the opening of the tube, so that the mass [23] lands exactly on the area of the head which had the duramater exposed, to ensure the trauma model has caused damage to the brain, 1 rat was put down for pathology examination with hematoxylin staining, the results were hemorrhage in the brain tissue, while other rats were treated according to post-craniotomy procedure protocols, lastly all wounds were sutured using zyde 5.0 after antibiotic ointment was administered. All surgical procedures were performed aseptically by adhering to the principle of sterility. After the procedure, all rats were treated in a recovery room with room temperature settings before returned to their cages.

### 3.2 CAPE administration

CAPE obtained from Sigma-Aldrich Pte. ltd with Reagan Number 10454-70-9 was prepared in saline solution and given through an intraperitoneal injection (IP) administered 30 min post-trauma at a dose of 10 mg/kg, then repeated daily for 7 days, the control group was given a placebo with the same set-up as the CAPE group.

### 3.3 Sample collection and examination

Blood is drawn 24 h before treatment which is used as the base value of each sample, then is also taken on day-4 and day-7 of treatment. All blood samples were examined with an 8-iso-PGF2 Rat (8-isoprostane) ELISA kit with Catalog No. MBS7606827 which was bought from Mybiosource.com.

### 3.4 Statistical analysis

The data is presented as mean ± SD. All data were processed and analyzed using Excel 2013 and SPSS version 23 (IBM Corp. Released 2015. IBM SPSS Statistics for Windows, Version 23.0. Armonk, NY: IBM Corp.). F2-IsoPs levels were analyzed with Independent T test. P-values less than 0.05 were considered statistically significant.

## 4 Results

All rats survived after trauma until a predetermined experimental time point was reached.

### 4.1 Characteristics of research subjects

Characteristics of Sprague-Dawley rats are shown in Table 1.

**Table 1:** Sprague dawley rat body weight data.

	Mice Body Weight (gram)	p-Value
**Mean**	**290.07**	**0.155**
**SD**	± **10.48**	

The homogeneity test of Sprague Dawley rats as a head injury model using the levene homogeneity test yielded a p > 0.05 was obtained, and it can be concluded that each rat's weight was not significantly different.

**Fig. 2:**
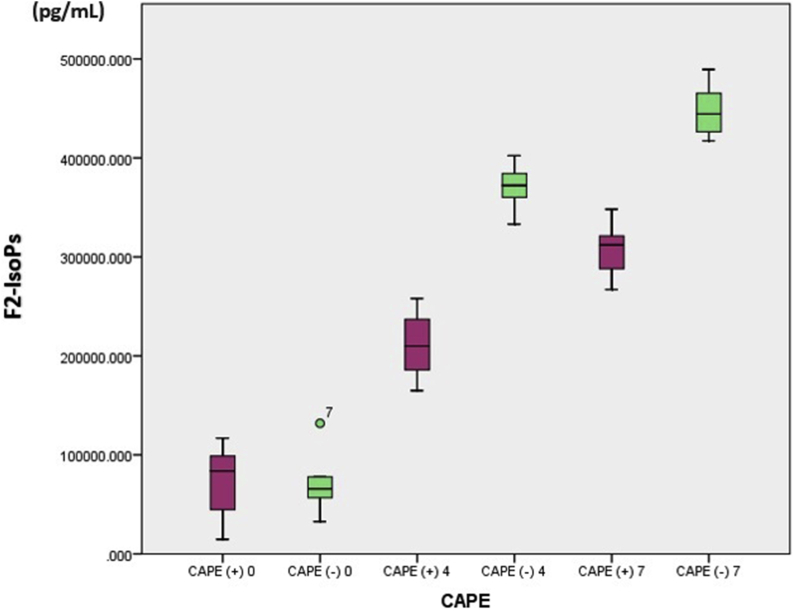
Daily F2-isoprostane serum levels in rats with and without caffeic acid phenylethyl ester (CAPE).

The average F2-IsoPs levels in rats with brain injury that were given CAPE compared to those not given CAPE on day 4 were 211210.376 ± 37559.206 pg/mL versus 370435.934 ± 25982.905 pg/mL (p < 0.05) and day 7 were 307346.562 ± 31119.798 pg/mL versus 448546.585 ± 29328.062 pg/mL (p < 0.05). The values were found to be significantly lower in the treatment group compared to the non-treated group (Table 2). On the seventh day, the F2-IsoPs levels in both groups were higher than on the fourth day. This finding can also be is clearly illustrated in the Boxplot graph below (Fig. 2):

**Table 2:** Levels of F2-isoprostane in rats with experimental brain injury.

Day-	CAPE Treatment	F2-isoprostane (pg/mL)		Mean difference	P-value
		Mean	SD		
0	(+)	71812.907	41519.641	−1201.702	0.963
	(−)	73014.609	36831.245		
4	(+)	211210.376	37559.206	−159225.56	<0.001
	(−)	370435.934	25982.905		
7	(+)	307346.562	31119.798	−141200.023	<0.001
	(−)	448546.585	29328.062		

**Abbreviations**: SD = standard deviation; Independent T-test with a p value < 0.05 was significant, CAPE: Caffeic acid phenyl ester.

## 5 Discussion

The results of this study showed a significant decrease in the amount of F2-IsoPs in the Sprague Dawley blood serum, in previous studies it was found that in cases of brain injury, an increase in oxidative stress would occur [1,4,24]. The oxidative stress that is formed is characterized by an increase in ROS (hydrogen peroxide, superoxide anions, and other free radicals) that is not matched by antioxidant defenses [4,25,26]. ROS is a normal substrate of aerobic metabolism derived from oxygen reduction, important for intracellular signaling systems under physiological conditions [1,27].

The reaction of the hydroxyl radical (OH) with unsaturated fatty acids produces lipid radicals that can react with oxygen molecules (O_2_) to form peroxyl lipid radicals. Peroxyl lipid radicals can take hydrogen from adjacent fatty acids to form lipid hydroperoxide (LOOH) and other lipid radicals. Alkoxyl and peroxyl radicals trigger lipid peroxidation chain reactions by removing hydrogen atoms. Lipid peroxidation is one of the main causes of cell damage. The process of fatty acid peroxidation mainly occurs in the phospholipid membrane. Various products are produced due to lipid peroxidation such as F2-IsoPs, MDA (Malondialdehyde), and F4-Neuroprostane [28]. In this study we analyzed the F2-IsoPs levels in Sprague Dawleys with brain injury.

CAPE and its metabolites, are efficient in detoxification of reactive oxygen species (ROS), for example, HOˉ and H_2_O_2_ and they also interact directly with reactive nitrogen species (RNS), as shown in Table 2 [[19], [20], [21]]. Quantification of F2-IsoPs is a sensitive index of neural oxidative damage, which can represent oxidation status occurring in the CNS [29]. At present, the detection of F4-NeuroPs and F2-dihomo-IsoPs is examined mainly in brain tissues and/or body fluids [1,4,30]. This process is in line with a study conducted on athletes who train in high altitudes for 2 weeks, found that F4-NeuroPs were detected in the athletes’ urine, this shows that the height factor can be related to the production of DHA (Docosahexaenoic Acid), through lipid peroxidation. As a main conclusion, exercise conducted at a moderate altitude increases F4-NeuroPs and F2-dihomo-IsoPs in relation to oxidative damage from CNS [1,4,31].

CAPE is a molecule with antioxidant and cytoprotective properties and plays a role in immunomodulation, is obtained from propolis extracted from honey bees [16,17]. At first propolis was known as a traditional medicine [16]. The ischemic process that follows a head injury causes accumulation of lactic acid due to anaerobic glycolysis, increases membrane permeability, and edema [1,2,26]. This is followed by cell membrane depolarization and excessive release of glutamate and aspartate neurotransmitters, activation of *N*-methyl-d-aspartate, α-amino-3-hydroxy-5-methyl-4-isoxazolpropionate and changes in voltage-dependent Ca^2+^ and Na^+^ channel [4,26]. Constant influx of Ca^2+^ and Na^+^ triggers the process of intracellular catabolism. Ca^2+^ activates lipid peroxidase, protease, and phospholipase which increase intracellular concentrations of free fatty acids and free radicals (ROS) [1,2,4,32].

## 6 Conclusion

From the results and description above, it can be concluded that the administration of CAPE in a rat model with brain injury can reduce the formation of F2-IsoPs as an indicator of oxidative stress in blood serum post-trauma.

## Author contribution

RAN, AAI, MH, AT, NAL, and PRI wrote the manuscript and participated in the study design. RAN, AAI, AT, NAL and PRI drafted and revised the manuscript. RAN, AAI, AT, NAL and MF performed head trauma treatment and surgery. AAI, MH, PRI and MF performed bioinformatics analyses and revised the manuscript. All authors read and approved the final manuscript.

## Registration of research studies

None.

## Guarantor

Rizha Anshori Nasution

## Funding

No funding or sponsorship

## Ethical approval

All procedure for Animal experiment has been approved by Ethics Commission Faculty of Medicine, Hasanuddin University, Number: 771/UN4.6.4.5.31/PP36/2019.

## Consent

This manuscript does not involve human participants, human data, or human tissue.

## Provenance and peer review

Not commissioned, externally peer reviewed.

## Declaration of competing interest

The authors declare that they have no conflict of interests
